# Effect of Environment on the Cognition of Older Adults: A Narrative Review

**DOI:** 10.3390/brainsci16050502

**Published:** 2026-05-02

**Authors:** José Miguel Sánchez-Nieto, Beatriz Hernández-Monjaraz, Víctor Manuel Mendoza-Núñez

**Affiliations:** Research Unit on Gerontology, FES Zaragoza, National Autonomous University of Mexico, Mexico City 09230, Mexico; cheverego@hotmail.com (J.M.S.-N.); beatrizhmonjaraz@hotmail.com (B.H.-M.)

**Keywords:** neighborhood characteristics, green space, walkability, aging in place, built environment

## Abstract

**Highlights:**

**What are the main findings?**

- Participation in intellectually stimulating activities, physical exercise, and a healthy diet, along with the mitigation of chronic stress, reduction in depressive symptoms, and attenuation of the adverse effects of air pollution, are proposed as plausible pathways linking neural processes and the environment.
- Environmental conditions at the micro-, meso-, and macro-levels potentially associated with cognitive maintenance in older adults have been described.

**What are the implications of the main findings?**

- These factors may be considered when designing cognitive maintenance programs for older adults across micro-, meso-, and macro-level environments.

**Abstract:**

Cognition in older adults may be influenced by environmental factors; however, the pathways linking environmental exposures and cognition remain unclear. The aim of this narrative review is to synthesize evidence on the association between the environment and cognition in older adults, integrating biological, environmental, and behavioral elements. Systematic reviews and original studies addressing this topic were identified in Web of Science, PubMed, and Scopus. The primary neural processes associated with maintaining cognition during aging are neuronal plasticity and compensatory scaffolding. Participation in intellectually stimulating activities, physical exercise, and a healthy diet; mitigation of chronic stress; reduction in the severity of depressive symptoms; and buffering against the adverse effects of air pollution are proposed as plausible pathways that may mediate the relationship between neural processes and the environment. In this context, environmental factors that affect cognition can be classified at three levels: (i) micro-level (family and home): social interaction with family members and indoor pollution; (ii) meso-level (community and services): social interaction, land-use diversity, transportation systems, environmental design, and urban green spaces; and (iii) macro-level (society in general and public policies): social representations of old age and aging (positive aging vs. ageism) and public policies aimed at improving pathways related to cognitive maintenance. Overall, the environment may influence cognition in older adults; however, the available studies show methodological and conceptual heterogeneity, inconsistent findings, and important gaps in knowledge.

## 1. Introduction

The global demographic transition presents a challenge that requires feasible strategies to ensure healthy aging, considering different sociocultural contexts. In this regard, the World Health Organization reported the following data on population aging: (i) between 2015 and 2050, the proportion of the world’s population over 60 years of age will almost double, from 12% to 22%; (ii) by 2020, the number of people aged 60 and over will exceed that of children under five; (iii) by 2050, 80% of older adults will live in low- and middle-income countries; (iv) the rate of population aging is much faster than in the past; and (v) all countries face significant challenges in ensuring that their health and social systems are prepared to address and take full advantage of this demographic shift [1,2].

Older adults are valuable sources of human and social capital and should be seen as a gerontological dividend, benefiting both their own development and that of individuals from different age groups. Three key elements to achieve this are the combination of healthy aging, productive aging after age 50, and counteracting ageism [3,4,5]. In this sense, older adults can continue to contribute to society through jobs that leverage their experience and knowledge in supervisory positions, as well as mentoring, participation in volunteer activities, home support, and more [6,7].

Healthy aging is defined as “the process of promoting and maintaining the functional capacity that allows well-being in old age” [2], specifying that functional capacity is determined by the individual’s intrinsic capacity, the environment in which they live, and the interaction between both. Among the components of intrinsic capacity is cognition, which allows information processing to interact with the environment [8,9]. Therefore, the aim of this review is to synthesize information on the effect of the environment on the cognition of older adults, linking biological, environmental, and behavioral elements.

## 2. Method

A search was carried out until 19 January 2026, on the scientific article platforms PubMed, Web of Science, and Scopus using Boolean operators (AND, OR) to combine terms such as “older” “elderly,” “social support,” “built environment,” “neighborhood environment,” “public policy,” “health policy,” “exercise policy,” “food policy,” “cognitive function,” and “cognitive decline,” among others. An example of the strategies used was (“built environment”) AND (“cognition” OR “cognitive function”) AND (older OR elderly). Systematic review and meta-analysis filters were applied. Studies examining the association between environmental factors and cognition in older adults were included. Environmental characteristics were categorized according to contextual level. At the micro-environmental level, studies addressing loneliness, interactions between spouses or family members, and the design and conditions of the immediate physical environment were considered. At the meso-environmental level, studies on social support networks, intergenerational relationships, land use, transportation systems, environmental design, and availability of green spaces were included. At the macro-environmental level, studies examining ageism and public policies aimed at promoting physical activity, social participation, and healthy diet, as well as reducing air pollution, were considered. Studies conducted among residents of long-term care facilities (nursing homes), and those focused on digital technology were excluded.

Full-text reviews were examined to identify principal findings and theoretical frameworks. Assessment of the methods and results of studies included within the systematic reviews revealed substantial heterogeneity and inconsistencies. To help interpret these findings, plausible key pathways linking environmental factors and cognition were identified, including cognitively stimulating activities, physical activity, diet, air pollution, depression, and stress. Based on these pathways, original studies were subsequently reviewed to synthesize evidence describing the relationships between the proposed key pathways, environmental exposures, and cognitive outcomes in older adults. Due to the non-systematic nature of this review, the possibility of bias in the selection and interpretation of evidence is acknowledged.

## 3. Environment Levels

**Figure 1:**
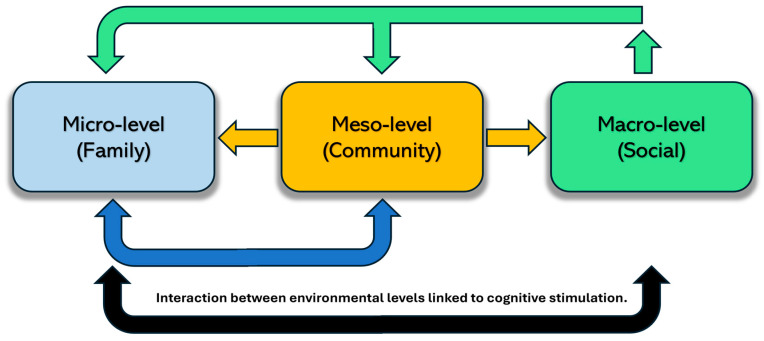
Micro-level: the characteristics of the home, the family, and their daily environment. Meso-level: the community, specific programs for older adults, spaces for leisure and recreation, generative programs (social participation), and health services. Macro-level: public policies, negative social representations of aging and old age (ageism), and recognition of the human and social capital of older adults for their own development. The arrows show the influence of the diverse levels of the environment.

The environment encompasses all external factors that form a person’s life context. It comprises three levels: (i) micro-level: the characteristics of the home, and family, (ii) meso-level: the community context, including programs for older adults, spaces for leisure and recreation, empowerment programs, and health services; and (iii) macro-level: society at large, public policies, social representations of aging and old age, institutional frameworks, and socioeconomic conditions, all of which influence both community and individual environments. Continuous interaction exists among these three levels (Figure 1) [10,11]. In this context, the environment may influence health, with certain conditions increasing the risk of disease [12], while others can promote a healthy lifestyle and improve health [13,14]. For this reason, the environment is a key element in promoting healthy aging, allowing older adults to maintain their autonomy and independence without needing to move to a long-term care facility [15].

Two theories attempt to explain how the environment can affect cognition: the biological theory of aging and the cognability theory. The biological theory of aging, developed from Bronfenbrenner’s ecological theory, proposes that well-being in older adults depend on the balance between environmental pressures and the level of individual competence. At the macro-environmental level structural characteristics of the country are considered, including the economic situation, the degree of urbanization, and crime rates, among other factors. The meso-environment includes aspects such as access to services and the perceived quality of the neighborhood, while the micro-environment encompasses home conditions, such as the organization of space, storage solutions, and access to outdoor areas. At all levels, active participation of older adults with family, friends, and community members is considered essential. When environmental pressures exceed the older person’s capabilities, a decrease in functional independence occurs [16].

The concept of cognability has been proposed to analyze how the neighborhood context structures opportunities and barriers to cognitive health in adulthood, with an emphasis on the meso-environmental level, which integrates characteristics of the built and social environment. Regarding the built environment, elements such as access to services, population density, street connectivity, the presence of spatial barriers, and the availability of green spaces are considered. The social environment encompasses factors such as community cohesion, social networks (including digital ones), sociocultural norms, and perceived crime and neighborhood disorder [17].

In this regard, several systematic reviews have reported an association between the community environment and cognition in older adults. Environmental characteristics linked to this outcome include population density, the diversity and accessibility of services, proximity to areas with high traffic volume, and the availability of green spaces. These factors have been assessed using both subjective and objective measures [18,19,20], however, these investigations have not delved into the mechanisms of this relationship or have focused solely on an environmental level of analysis, failing to identify the neural mechanisms that can promote cognitive maintenance, the key pathway that can influence these mechanisms, and the micro-, meso-, and macro-levels of environmental conditions that can affect these activities.

## 4. Cognition in Older Adults

Cognitive change during aging is a complex process: some functions increase while others decline. Cognition related to knowledge of the world and automatic processing, such as semantic memory, can continue to improve even after age 70. Conversely, cognitive abilities associated with acquiring new knowledge and deliberate processing, primarily attention and episodic memory, tend to decline as people age, especially after the age of sixty-five [21].

The decline in cognition during aging is not uniform. Working memory and processing speed begin to decline around age 30, while the decline in episodic memory becomes noticeable around age 60 [22]. Between the ages of 18 and 60, processing speed declines by one z-score, while episodic memory declines by 0.4 z-scores [23].

When cognition declines significantly, the person loses the ability to make decisions relevant to them (autonomy), and it can even affect the ability to perform basic and instrumental activities of daily living (independence), progressing to dementia, whose pathophysiological mechanisms depend on the type of dementia, among the most frequent are vascular dementia and Alzheimer’s disease [24].

In contrast, Mild Cognitive Impairment (MCI) can be a precursor to dementia, characterized by a greater cognitive decline than expected during normal aging, but the person maintains their autonomy and independence. Tools like the Mini-Mental State Examination (MMSE) and the Montreal Cognitive Assessment (MoCA) help detect MCI; they use cutoff scores that consider a person’s age and education level and are used in studies to assess global cognition. There are multiple causes of MCI, including altered blood pressure, high glucose levels, depression, and insufficient cognitive stimulation, among others. Typically, MCI is considered a potentially reversible condition that may be influenced by promoting a healthy environment across the micro-, meso-, and macro-levels [25,26].

Cognitive decline during aging is normal and no specific activities have been shown to stop this process entirely [27]; however, certain cognitive maintenance is possible to prevent the person from losing their autonomy and independence [28]. The biological mechanisms with the strongest empirical support that explain the maintenance of cognition during aging are brain plasticity and compensatory scaffolding [29,30].

Plasticity refers to the brain’s capacity to undergo lasting structural changes in response to environmental demands that are not met by the organism’s current functional capacity. This can be promoted through exposure to novel, complex, and sustained experiences. According to Lindenberger & Lövdén, structural changes emerge when there is a prolonged mismatch between an individual’s functional capacities and environmental demands, forcing neural reorganization. In this sense, interventions based on complex learning, environmental enrichment, cognitive training, motor skill acquisition, and social participation represent potential strategies for inducing brain plasticity during aging [29,31].

The Scaffolding Theory of Aging and Cognition, proposed by Park and Reuter-Lorenz, posits that scaffolding is a compensatory process by which the aging brain recruits additional or alternative neural networks, particularly in bilateral frontal regions, to counteract age-related decline and preserve cognition. This mechanism can be promoted through participation in activities that increase cognitive demands, such as learning complex and novel skills, deliberately using cognitive strategies to optimize performance on specific tasks, and engaging in socially challenging interactions that require planning, decision-making, and executive control [30,32].

In contrast, age-related cognitive decline has been linked to multiple interconnected biological mechanisms, most notably oxidative stress, and inflammaging. Increased oxidative stress causes cumulative damage to lipids, proteins, and neuronal DNA, while systemic inflammation and neuroinflammation promote sustained microglial activation and the release of pro-inflammatory cytokines that disrupt synaptic function. These processes are accompanied by a decrease in neurotrophic factors, a reduction in dopaminergic neurotransmission, impaired white matter integrity, and cerebral vascular changes, which contribute to cortical and subcortical atrophy, as well as disruption of neuronal connectivity and the efficiency of cognitive networks. Taken together, these mechanisms converge on reduced efficiency of neural processing and contribute to the progressive decline of memory, attention, and executive functions during aging [30,32].

## 5. Key Pathways Supporting Cognitive Maintenance

**Figure 2:**
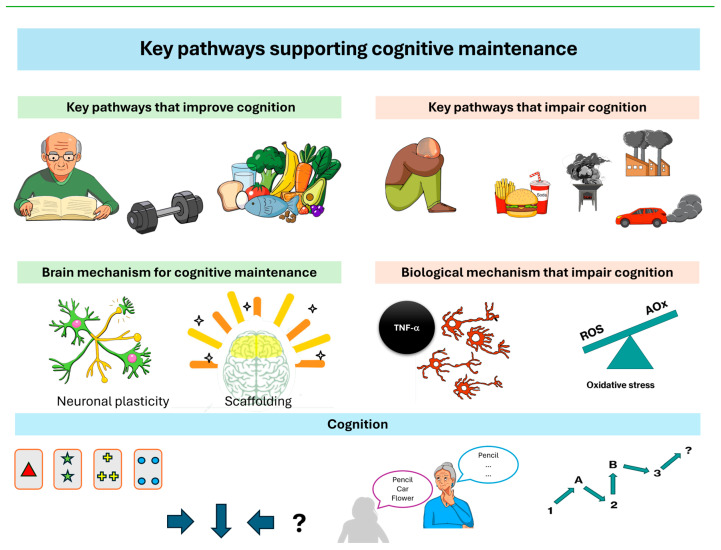
Key pathways supporting cognitive maintenance. On the left are the elements that can promote cognitive maintenance, and on the right, those that impair it. At the top level are behaviors and environmental factors; those that promote cognitive stimulation, exercise, and a healthy diet, while those that negatively impact it are isolation, depression, stress, sedentary lifestyle, unhealthy diet, and air pollution. At the middle level are brain mechanisms; those that promote cognition are plasticity and neuronal scaffolding, while those that negatively affect it are inflammation and oxidative stress. At the bottom level are the consequences of both, identified through neuropsychological evaluation.

Biological changes in the brain that occur during aging may be related to cognitive performance. Both beneficial and detrimental trajectories may be influenced by modifiable factors, some of which help explain how the environment is associated with cognition [30]. Factors that may contribute to maintaining cognitive function include engagement in intellectually stimulating activities, regular physical activity, and adherence to a healthy diet. In contrast, depression and chronic stress, unhealthy dietary patterns, and exposure to air pollution have been associated with poorer cognitive outcomes. We identify these factors as key pathways supporting cognitive maintenance, which are described below (Figure 2).

### 5.1. Intellectually Stimulating Activities

Cognitive training is based on standardized tasks to enhance a specific cognitive process; multiple studies have reported positive results [33,34,35,36], but with insignificant effect on tasks other than the exercises used in intervention programs [37,38]. Given these considerations, it has been suggested to develop mental stimulation programs centered around learning practical activities, such as using new technology for work or household tasks, or acquiring knowledge to care for other older adults [39,40].

Similarly, several observational studies have suggested that social participation, leisure activities, and intergenerational relationships contribute to cognitive maintenance [41,42,43,44]. Intervention programs based on intellectually stimulating activities such as learning photography or theater, creating products, or receiving support in academic activities, have been associated with positive effects on cognition [45].

Cognitive stimulation therapy is an intervention designed for people with MCI or dementia in its initial stages. Studies have demonstrated that it slows the rate of dementia-related decline. It primarily involves fostering social interaction through activities such as talking about the past, stimulating the senses, and practicing empathetic listening that validates subjective experience rather than objective facts [46].

Several intellectually stimulating activities can promote cognitive maintenance; the key is that they meet certain conditions. In line with what is expected to foster neuronal plasticity and compensatory scaffolding, as well as what has been reported in cognitive maintenance programs, the following characteristics are proposed for activities that can contribute to cognitive maintenance: (i) the use of different cognitive processes; (ii) the promotion of self-initiated processing; (iii) the performance of diverse tasks; (iv) the learning of new tasks; and (v) adjusting the difficulty of the task so that participants maintain a high level of effort. Some examples of activities with these characteristics are learning another language, using a computer, theater, teaching, among others [31,32,47,48,49].

### 5.2. Physical Activity and Exercise

Physical activity has been proposed as one of the main factors supporting cognitive maintenance [50,51,52]. Physical activity supports cognition by improving the production of neurotrophic factors, neurotransmitters, and hormones. It promotes various mechanisms associated with synaptic plasticity, neurogenesis, angiogenesis, and autophagy. It also is associated with reduced psychological stress, inflammatory cytokines, and oxidative stress [53,54]. In contrast, several studies have demonstrated the relationship between physical activity, diet, and sleep with a reduction in the probability of developing MCI or delaying the onset of clinical symptoms of Alzheimer’s [55,56,57].

In this regard, systematic reviews indicate that moderate-intensity aerobic exercise performed 3 to 5 days per week, complemented by strength training 2 to 3 times per week, and maintained during interventions of 8 to 52 weeks, is associated with significant cognitive improvements in older adults [51].

### 5.3. Diet

A healthy diet may act as a protective factor for cognition and can delay the onset of MCI or dementia. Increasing the consumption of vegetables, fruits, and fish can provide essential nutrients such as folate, trace elements, vitamins D, E, and B12, and antioxidants. These nutrients can protect the brain from damage caused by inflammation, oxidative stress, and vascular damage, as well as consumption of saturated fats and sugar can contribute to cognitive decline [58,59,60]. Furthermore, they increase the risk of chronic diseases such as diabetes mellitus or hypertension, which are risk factors for MCI [61]. Based on the above, a diet based on the consumption of fruits, vegetables, legumes, whole grains, fish, and unsaturated fats is associated with a lower risk of brain damage.

### 5.4. Psychological Stress and Depression

Psychological stress, primarily chronic stress, is a factor linked to increased cortisol levels. Several animal and human studies suggest a relationship between elevated cortisol and impaired cognitive performance. Cortisol exacerbates damage caused by oxidative stress and the production of β-amyloid peptide, affecting the hippocampus and frontal lobe [62]. Furthermore, it increases the risk of sleep disorders, depression, and vascular diseases, which in turn affect cognition [63,64,65].

Studies recommend that depression symptoms are a risk factor for MCI and dementia [66]. In this regard, older adults with depression are more likely to have a lower volume of gray matter, particularly in the hippocampus and frontal lobes. Depression can increase oxidative stress and inflammation, reduce neuronal plasticity, and increase apoptosis [66,67,68,69].

### 5.5. Air Pollution

Exposure to air pollutants such as particulate matter (PM), nitrogen dioxide (NO_2_), and ozone (O_3_) has been associated with poorer cognitive performance and a higher likelihood of decline [70,71]. PM causes the excessive production of reactive oxygen species (ROS). These species can lead to DNA damage, endoplasmic reticulum stress, inflammatory responses, atherosclerosis, and airway remodeling, contributing to increased susceptibility to and exacerbation of various diseases and infections. It primarily affects cardiovascular health, increasing the likelihood of strokes [72]. They can cross the blood-brain barrier through the bloodstream and accumulate in the brain for a long time, increasing levels of pro-inflammatory cytokines and oxidative stress, which facilitates the formation of amyloid plaques, which are related to Alzheimer’s disease [73,74].

## 6. Relationship of Different Environmental Levels with Cognition

**Figure 3:**
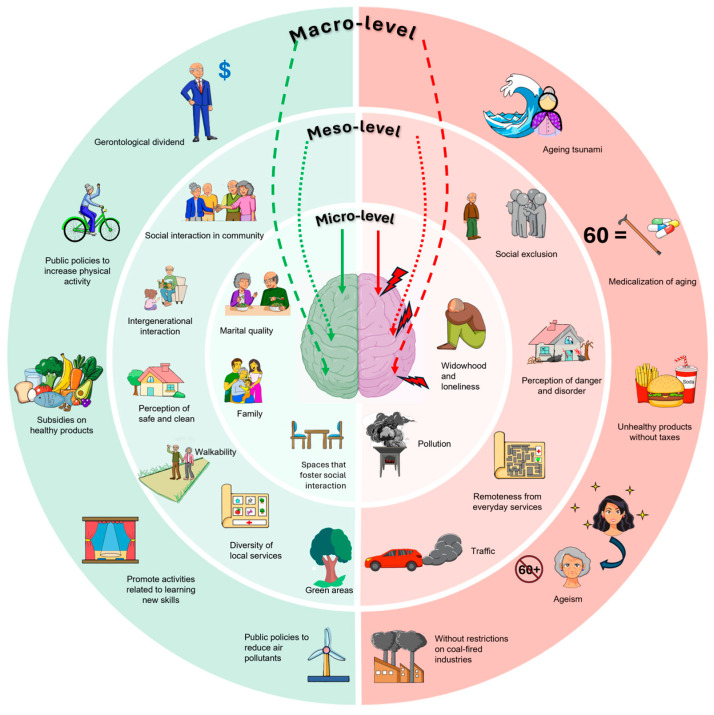
Influence of the multilevel environment on the cognition of older adults. An ecological model of the environment’s effect on cognition is shown. Potentially favorable factors are presented on the left, and those with a possible negative impact are on the right. At the macro-level, structural and public policy factors are included. These could involve a focus on recognizing the older adult population as the gerontological dividend and policies that promote healthy lifestyles and reduce pollution. In contrast, there is a public policy view that considers the increase in older adults as a demographic tsunami, coupled with ageism due to the lack of recognition of the human and social capital of the older adult population and the medicalization of aging without considering a healthy aging approach. At the meso-level, the characteristics of the community environment are represented, where social interaction, peer support, walkability, availability of services, and green spaces are associated with favorable effects, while social exclusion, insecurity, physical disorganization, and lack of services are associated with adverse effects. At the micro-level, factors from the immediate environment are included, such as marital relationships, family support, and housing design that fosters social interaction, as opposed to loneliness, widowhood, and indoor air pollution. At the center, the brain represents the influence of these factors on cognition.

The environment, at the micro-, meso-, and macro-levels, has been associated with cognitive performance in older adults; however, the key pathways linking these environmental dimensions to cognition have not been fully integrated. In this context, we propose a set of factors potentially modulated by the environment that may directly influence cognitive functioning, including intellectually stimulating activities, physical exercise, a healthy diet, air pollution, depressive symptoms, and chronic stress. Considered together, these elements provide a conceptual framework for articulating the relationships between the environment and cognition. The following section describes the main environmental indicators reported in the literature and examines how the proposed factors, as key pathways, may help explain these associations (Table 1 & Figure 3).

### 6.1. Environment Micro-Level (Family)

The microenvironment can affect cognition due to the type of behavior or activities that older adults perform daily in their homes. In this regard, social factors, primarily contact with a partner or children, as well as physical factors, such as indoor pollution, temperature, room layout, or decor, influence cognition [75,76,77].

#### 6.1.1. Social Interactions in Family

Research has suggested suggests that growing up in a stimulating environment may support cognitive development during childhood [78]. One of the most widely used instruments for assessing environmental conditions that can stimulate a child’s cognition is the Home Observation for Measurement of the Environment (HOME). Recently, it has been adapted for older adults, observing that a greater variety of stimulation is related to higher cognitive performance. Some signs of this variety are living with pets, family, or friends, as well as taking part in cultural events; still, it’s important to consider what these relationships are like [79]. The following is an analysis of the effect of relationships with partners and children on cognition.

The relationship between marital satisfaction or quality and cognitive performance is inconsistent. Marital satisfaction is a subjective assessment of how happy a person is in their marriage, while marital quality includes elements such as affection, communication, cohesion, and consensus, among others. Studies have suggested that people with higher marital satisfaction or quality tend to perform better cognitively, indicating that less marital tension has been linked to improved cognitive abilities. The proposed biological link to explain the positive relationship between marital satisfaction and cognitive performance is the reduction of psychological stress [75]. However, given the inconsistencies across studies, other lifestyle-related factors, including those proposed here as key pathways linking the environment and cognition, may also influence cognitive performance.

It has been reported that people with a partner with MCI have a higher probability of developing MCI themselves. Three explanations have been proposed: (i) the impact of caregiver stress experienced by the spouse; (ii) partner selection based on similar characteristics, some associated with MCI, such as low educational attainment or similar age; (iii) the influence of shared environment and lifestyle, for example, a sedentary lifestyle, a diet that increases the risk of metabolic diseases, or few intellectually stimulating activities [80].

Widowhood is a risk factor for sudden cardiac death, particularly in the first four years. This relationship may be explained by the way grief is experienced after losing a partner, which can manifest as depression or a decrease in activities, including physical exercise and those that contribute to mental stimulation [81].

Relationships with adult children also influence cognitive function. In particular, bidirectional financial support has been found to be associated with better cognitive performance in older adults. A positive relationship is also reported between sharing household chores or childcare, helping with schoolwork, or teaching skills and preventing cognitive decline. In contrast, excessive (unnecessary) instrumental support for older adults has been associated with a higher likelihood of developing MCI [76]. Also, in rural areas, when older adults live alone because their children have left home, the risk of heart attack and suicidal thoughts increases [82].

Several studies have identified loneliness as a risk factor for increased MCI. However, longitudinal evidence examining the long-term effects of loneliness remains limited, particularly in populations with fewer socioeconomic resources. In addition, most studies rely on global cognition scores, with relatively little attention to specific cognitive domains. In this context, the proposed factors, such as depressive symptoms, may contribute to reduced participation in intellectually stimulating activities, and decreased physical activity, may also be considered as potential pathways that could help explain the increased risk of MCI associated with loneliness [83,84].

Considering the analysis above, psychological elements such as a sense of positive emotional connection, autonomy, or a sense of purpose in life are factors that motivate people to start and maintain a lifestyle [85,86], however, the effect on cognitive maintenance depends on the activities undertaken during social interaction. Activities that promote healthy aging, shared with the family and in which the older adult plays an active role, such as cognitively stimulating activities (board games, conversations about new topics, written assignments, computer use, among others), physical exercise, and a proper diet, can have an impact on cognitive function [87]. Even some solitary tasks that meet the characteristics of intellectually stimulating activities, described in the Section 5.1, can positively affect cognitive performance [88].

#### 6.1.2. Physical Elements of the Environment

Characteristics of the microenvironment have been proposed as being associated with cognitive functions such as perception, attention, memory, and executive functions. Among the main ones are pollution and temperature; others have a particular impact on people with MCI, such as space distribution and decoration [77].

Currently, in countries like China and India, over 45% of homes continue to use solid fuels such as wood, coal, kerosene, crop residues, or manure for cooking and heating, in addition to incense sticks and mosquito coils. The use of these products increases the likelihood of developing myocardial infarction, even after controlling variables such as age, education level, and socioeconomic status [89,90]. This may be due to the increase in PM [91]. In addition, air pollution has been linked to depression, sleep disorders, and hypertension, each of which constitutes a risk factor for MCI in older adults [92,93,94]. Good ventilation may help reduce the cognitive effects of pollution; a study in China found that ventilating the house 6 to 8 times per week is associated with reduced the risk of MCI [95].

A study found a link between having plants and flowers indoors and improved cognition. This may be due to their relaxing effect and the reduction in indoor air pollution. However, this effect may also be masked, as participants in the study also took a course on plant care. Therefore, the association with improved cognition is more likely due to this activity rather than the reduction of stress or indoor pollution. However, additional studies are necessary [96].

Extreme temperatures are associated with cognitive performance in older adults; in particular, elevated temperatures lead to poorer cognitive performance [97]. In a study that monitored house temperatures and asked older adults about their perceived level of attention while performing daily tasks, researchers found that a temperature between 20 and 24 °C was associated with better levels of attention [98]. In contrast, in an experimental study in which 68 older adults performed cognitive tests at 24 °C or 32 °C, lower cognitive performance was associated at elevated temperatures, only in those participants who did not perform physical exercise [99].

The environment can help maintain cognition by facilitating interaction between people. Spaces encourage eye contact, for example, by avoiding walls between rooms or placing chairs around the table to allow people to see each other face-to-face [100]. Spaces that facilitate movement between places in the house can also promote social interaction [101]. Social interactions can motivate people to engage in activities, but the type of activity performed will depend on the influence it has on cognitive maintenance.

Other environmental elements may have minor impact on the cognitive stimulation of healthy older adults; however, they can improve the functionality of people with MCI or the initial stages of some dementias. Higher contrasting colors and textures can make it easier for them to recognize personal objects, such as their room, bed, plate, etc. [102,103]. Familiar decorative objects can encourage memory [104]. In contrast, similar textures or colors between objects can make it difficult to differentiate them, for example, a table and a plate of the same color. Also, textures with crossed or uneven lines can facilitate the development of hallucinations [105,106].

Research on the effects of the physical home environment on cognitive functioning remains in an early stage of development. Evidence examining indoor air pollution and temperature is limited, and most available studies rely on cross-sectional designs, restricting causal inference. Further research across diverse geographic and socioeconomic contexts is needed to better understand the potential impact of these environmental conditions. In addition, intervention studies aimed at improving indoor air quality and thermal comfort could help clarify their role in cognitive health among older adults.

Similarly, evidence regarding the distribution and organization of home spaces is scarce. Future studies should examine whether these environmental characteristics facilitate engagement in intellectually stimulating activities, promote physical activity within or outside the home, or contribute to reducing depressive symptoms and stress in older adults. Addressing these gaps may help elucidate the pathways linking the home environment to cognitive functioning and inform subsequent areas of research.

### 6.2. Environment Meso-Level (Community)

Research on the meso-environment is primarily conducted at the community level. At this level, the analysis of the association between environmental characteristics and cognitive functioning typically focuses on both community social interactions and features of the built environment. The built environment refers to spaces modified by humans to facilitate daily activities such as living, working, and recreation. Among the characteristics of the built environment that have been associated with cognitive functioning are land-use patterns, transportation systems, environmental design, and access to open spaces [107,108]. The following section describes the relationship of community social interactions and the built environment with cognitive functioning.

#### 6.2.1. Social Interaction in Community

Community-level social interaction is commonly examined through the study of social networks, which comprise both structural and functional components. Structural elements refer to the configuration of social relationships surrounding the individual and include indicators such as network size, frequency of contact, and participation in social activities. These activities encompass social engagement, meeting with friends, attending community events, and participating in work-related, recreational, or volunteer activities. Functional components, in turn, correspond to perceived social support, defined as an individual’s perception of the availability of assistance from their social network, which may be emotional, instrumental, or informational in nature [109,110]. In contrast, social exclusion has been conceptualized as the process by which individuals or groups are marginalized from participation in society, limiting their access to material resources and services, social relationships, community infrastructure, transportation, and civic and sociocultural activities [111].

Several systematic reviews including observational studies, both cross-sectional and longitudinal, suggest that structural characteristics of social networks, particularly larger network size, higher frequency of contact, and greater participation in social activities are associated with global cognition in older adults. However, findings are inconsistent, which may be partly explained by heterogeneity in the operationalization and measurement of structural network characteristics. In addition, the specific effects of contact frequency and network size remain insufficiently clarified; this could be addressed by incorporating the evaluation of the key pathways proposed in our conceptual framework [109,110,112]. Additionally, reviews of experimental studies focusing on participation in social activities, including physically active and cognitively stimulating activities, have also reported positive effects on cognitive performance, although substantial heterogeneity exists in the type and characteristics of the activities evaluated [113].

Regarding functional components, some cross-sectional and longitudinal studies have described a small positive association between emotional support and cognitive performance. However, other studies have not found significant associations and, in some cases, have reported negative relationships. These discrepancies may be attributable to variability in the definition and measurement of social support, highlighting the need to distinguish qualitative characteristics of emotional support, such as affection, trusting, or positive relationships. For other types of support, including instrumental or informational support, findings are even more inconsistent, and the available evidence does not support a clear association with dementia. Furthermore, negative interactions within social networks, such as rejection, unsolicited advice, or lack of support, have been insufficiently explored, despite their potential association with poorer cognitive performance [109,114].

Conversely, social exclusion has been associated with poorer cognitive performance and an increased risk of MCI. A systematic review including observational studies reported that economic exclusion is associated with poorer cognitive performance, although some studies have paradoxically found that individuals with fewer economic resources exhibit a lower risk of cognitive decline. Consistently, several studies have linked loneliness, social isolation, smaller social networks, reduced contact with family and friends, and lower participation in social activities with poorer cognitive performance. However, these findings are not uniform, and some studies have not identified significant associations, which may reflect heterogeneity in the assessment of social indicators and cognitive outcomes [111].

In addition, intergenerational interaction in community settings may also represent a relevant factor for maintaining overall health, reducing ageism, and promoting cognitive maintenance, particularly those related to cognitively stimulating activities and physical activity [115]. In this context, interventions have been evaluated across different settings, including community centers, long-term care facilities, educational institutions, and recreational spaces, incorporating activities such as health promotion, storytelling, computer training, physical activities, and museum visits, among others. Overall, findings suggest that these interventions may reduce symptoms of anxiety and depression, decrease age-related stereotypes, and enhance generativity and social cohesion [116,117,118]. Some studies have examined the characteristics of intergenerational activities that may be beneficial for enhancing social participation and promoting physical and psychological well-being among participants across the lifespan [115]. Although such activities may influence cognition in older adults, most studies have not directly assessed this outcome, and the available evidence is heterogeneous and limited, precluding consistent conclusions [116,117,118].

Regarding the effects of intergenerational interaction on cognition, the Experience Corps program has been particularly notable. This initiative involves older adult volunteers providing literacy support to elementary school students. Studies derived from this program have reported improvements in memory, particularly among participants with poorer baseline executive function performance, as well as better subjective perceptions of cognition. Additionally, other studies have evaluated older adults participating in reading-to-children programs, in which a slower decline in memory performance and reduced hippocampal volume loss have been observed compared with control groups. Nevertheless, the evidence remains limited and highly heterogeneous in terms of intervention design and outcome assessment methods. Furthermore, no studies were identified that specifically examine how modifications in physical features of the community environment may influence intergenerational social interaction and, in turn, cognitive performance in older adults [116,117,118].

Studies examining the relationship between social interaction and cognition in older adults present several methodological limitations. These include the lack of differentiation between types of social ties within networks, such as family members, friends, or community members, as well as the absence of distinction between cognitively stimulating social activities and those primarily involving physical activity. In addition, the effects of negative social interactions on cognition have been insufficiently explored. Several studies also fail to adequately control for potential confounders, such as depressive symptoms, physical activity level, and alcohol or tobacco use. These limitations are compounded by heterogeneity in the measurement of social indicators and the predominant use of global cognition instruments to assess cognitive outcomes, which restricts the identification of domain-specific associations [109,110,111,113,114,116,117,118]. Addressing these methodological issues would allow a more precise understanding of the pathways through which community-based social interaction may contribute to the cognitive maintenance in later life.

#### 6.2.2. Land Use Patterns

Land use patterns refer to the spatial distribution of human activities, for example, their use for residential, recreational, or commercial purposes (this also includes green areas, but these will be addressed in the Section 6.2.5 on open spaces). Areas with a higher mix of land uses are associated with a lower likelihood of MCI [119,120] and better memory [121]. In contrast, living in an area with low residential density, where there may be fewer services, was associated with a higher probability of developing MCI [122].

Not all land uses will have the same impact on cognitive function, nor on the same populations. Distance from everyday services such as convenience stores is associated with poorer cognitive performance and a higher likelihood of developing dementia in populations of all economic strata; however, distance from healthcare centers only impacts middle- and low-income populations [123]. Another study found that in people under 80, a greater mix of land uses was associated with better cognition, while in people over 80, proximity to a community center had a positive impact on cognition [124]. It has also been reported that the proximity of community centers improves cognition in white people, but not in black and Hispanic people [125].

The availability and diversity of local services may also play a role in cognitive functioning. Services that promote active leisure have been associated with better performance in older adults [124]. A longitudinal study found that proximity to civic and social organizations that promote participation was associated with improvements in cognition; likewise, proximity to artistic organizations, museums, and recreation centers was associated with stability in cognition [17].

Participation in activities within the surrounding environment may also represent a relevant factor associated with cognitive functioning. In a study by Guo et al. [126], better cognitive performance was found in areas with greater access to libraries, but only among highly educated individuals. Meanwhile, another study observed a positive relationship between processing speed and time spent shopping, but not with destination density [127]. Similarly, in areas with less availability of services, people who participated in more activities in their community, such as cultural classes, meetings, attending community centers, and participating in sports and gardening activities, were less likely to experience MCI [128].

In contrast, access to services can improve or impact overall health, which would indirectly affect cognitive function. Living within 500 m of fruit and vegetable shops is associated with a lower risk of developing dementia [129], while the proximity of fast-food establishments is associated with poorer cognitive performance [17]. It has also limited access to healthy food linked to greater cognitive decline [130].

#### 6.2.3. Transportation System

The transportation system refers to the physical infrastructure and services that provide spatial links or connectivity between activities. Elements that have been associated with cognitive function in older adults include street integration, connectivity, walkability, transportation lines, and proximity to major transportation routes [108].

Street integration measures the number of choices a person has to access locations within a defined system; lower integration indicates more winding paths or dead ends. Connectivity refers to the number of paths, streets, or nodes directly connected to each street or node in the road network. In older adults, greater street connectivity and integration may be associated with better cognitive performance [131]. It was also in places with more bus lines and employment services; cognitive decline is slower [132].

Roads with a higher number of intersections and a greater proportion of land dedicated to retail trade were associated with better processing speed [133]. Whereas, in places with a lower density of intersections, older adults have lower cognitive performance [134]. In individuals carrying APOE ε3 or ε4, the positive effects of intersections on cognition were not observed [133].

Walkability can be particularly beneficial for people with poorer levels of education. Walkability refers to the density of intersections and the shorter distance to the nearest public transport stop. Greater walkability may contribute to reduce the distance gap in cognitive performance between people with poorer levels of education and those with higher levels of education, while neighborhoods that increase social isolation widen that gap [135].

In contrast, evidence has suggested that residential proximity to major traffic roads may be associated with non-Alzheimer’s dementia; the effect is found at 50 m, although the negative effect occurs at a greater distance in large cities [136], even at 150 m in the case of highways [137]. Similarly, living 100 m away from main streets is associated with poorer cognitive performance [138,139]. Furthermore, living near a busy area affects older adults with low levels of education more [139].

#### 6.2.4. Environmental Design

Environmental design refers to the aesthetic, physical, and functional qualities of an area. In research on cognition, cleanliness and safety are the main factors studied. Objective elements of cleanliness (e.g., litter, graffiti, noise, and unpleasant odors) which have been associated as objective indicators of danger in an area (e.g., murders, rapes, or assaults), are not associated with cognitive performance [140,141].

In contrast, design indicators evaluated subjectively, that is, people’s perceptions of their environment are related to cognitive function in older adults. In environments perceived as more dangerous, older adults exhibit lower cognitive performance [140,141]. Environments perceived as having greater physical disorder (e.g., graffiti, garbage, empty houses, and crime) have also been associated with poorer performance in episodic memory; this may be explained by increased stress in older adults [142,143].

#### 6.2.5. Open Spaces

Open spaces include green spaces, blue spaces, and areas with little or no vegetation that serve as spaces for social interaction. Green space is a general term encompassing various forms of urban landscapes, such as isolated trees, green walls, open lawns, and dense forests with or without access. Blue spaces are areas where water predominates; their functions are like those of green spaces [144,145].

A meta-analysis of five studies found that living near a green area was associated with a reduced the risk of dementia (OR = 0.94, 95% CI 0.92, 0.96) [19]. Longitudinal studies have suggested that living within 300 m of a green space is associated with reduced the risk of cardiovascular events, vascular dementia, and death. The impact of green spaces on reducing the likelihood of strokes was greater in those who had previously suffered a stroke [146,147]. Greater tree exposure has also been associated with a lower likelihood of developing dementia by 14% over an 11-year period [148]. The protective effect of green spaces in preventing cognitive decline has also been reported in a longitudinal study that analyzed people from 11 to 76 years old [149].

A study found that people living within 500 m of a green space have better cognitive performance compared to those living farther away. It also observed that people living near green spaces are less likely to develop MCI [150]. Living near blue spaces may also reduces the risk of dementia linked to cognitive health benefits [151]. Moreover, proximity to a green area mitigated the negative cognition effects from living near a road [137].

The protective effect of green spaces may not be present in all cases. One study reported a protective association between green spaces and cognition in older adults between 65 and 79 years old, unless they carry the APOE ε4 gene [152]. Another study found that access to recreational spaces had better cognitive performance, but only in men, not in women [153]. In another study, the protective effect of green spaces on cognition was only observed in women from low-income backgrounds [149].

Systematic reviews examining built environment characteristics, including land use, transportation systems, environmental design, and open spaces, have suggested an association with cognitive performance in older adults. However, the available evidence remains of moderate strength, is derived primarily from observational studies, and findings across studies included in these systematic reviews are inconsistent. In addition, several attributes of the built environment have been investigated in only a limited number of studies. Even when similar features are assessed, their operationalization and measurement vary considerably across investigations, limiting comparability [18,19,108,145].

Furthermore, the spatial scales used to define the community environment vary considerably across the literature, with buffer sizes ranging from 200 m to 1600 m, which may contribute to the heterogeneity of findings. There is also a paucity of evidence regarding specific architectural design features, such as lighting, sidewalk width, signage, access to public restrooms, and the availability of resting areas, all of which may influence cognitive performance in older adults. In addition, there is limited integration between objective and subjective environmental measures. Objective indicators typically capture the physical attributes of neighborhoods, whereas subjective assessments may reflect individual perceptions and preferences, which could be more closely related to actual patterns of space use [18,19,108,145].

Moreover, most existing studies rely on global measures of cognitive functioning, highlighting the need to incorporate assessments of specific cognitive domains. Finally, further research is needed to examine mediating mechanisms, including the key pathways proposed in this review, as well as potential moderators such as age, sex, and educational attainment [18,19,108,145].

### 6.3. Environment Macro-Level (Social)

The macroenvironmental elements that can affect cognition are primarily related to social representations of aging (ageism) and public policies. Ageism refers to prejudices (feelings), stereotypes (thoughts), and discrimination (actions) directed at a person based solely on their age, whether self-inflicted, interpersonal, or institutional [154]. In contrast, public policies for healthy aging, primarily aimed at promoting social participation and exercise and reducing the consumption of processed products and pollution, influence cognition [155,156,157].

#### 6.3.1. Ageism

Self-inflicted ageism is the internalization of stereotypes and prejudices about old age, which affect an individual’s self-perception, expectations, interests, and actions. Self-inflicted ageism not only affects cognitive function but also overall health [158]. Some of these beliefs, such as the idea that all older adults have bad memories, cannot learn new things, cannot meet new people, and are simply a burden on society, among others, can limit a person’s ability to engage in intellectually stimulating activities, exercise, or maintain a healthy diet [159].

A systematic review of longitudinal studies suggests that a positive perception of aging is associated with better health outcomes, including lower obesity, greater longevity, reduced depression, and better cognitive performance. Several mediating pathways have been proposed, including physical activity, social participation, and reductions in depression and stress. However, there is a need for further studies examining these relationships using robust mediation models. In addition, most of the included studies were conducted in high-income countries, limiting the generalizability of the findings to other socioeconomic contexts [160].

Ageism in interpersonal relationships manifests in interactions with older adults, as well as in comments and behaviors directed toward them. Negative interpersonal stereotypes can foster self-inflicted stereotypes and generate discriminatory behaviors that limit developmental opportunities and cognitive stimulation. Conversely, positive stereotypes can promote prosocial behavior toward older adults [161], however, such support can limit opportunities for social participation and activities, affecting cognitive performance, when a person is overprotected simply because they are old [162,163], therefore, it is important to consider support that enhances autonomy and independence, and not just welfare-based assistance.

Institutional ageism encompasses laws, regulations, policies, and practices that unfairly limit opportunities based on age. It includes conscious and overt actions by individuals within the institution, although more often it involves repetitive behaviors without any analysis of their effects and implications. The assessment of Institutional ageism is based on the outcomes of the institution’s activities and can affect employment, financial support, access to media, research, healthcare, and other areas [164]. For example, in healthcare, frailty is emphasized, medical care or procedures can be denied, unnecessary treatments can be administered, and other factors can be exploited. This affects not only cognitive performance but also quality of life and life expectancy [165].

Overall, the evidence suggests that negative age-related stereotypes may influence cognitive functioning in older adults. However, the available findings are inconsistent, and most studies have relied on global measures of cognition, limiting the understanding of more specific cognitive effects. In addition, there is a paucity of research examining potential mediators and moderators of this relationship in a systematic manner. Proposed mechanisms include physical activity, social participation, and reductions in depressive symptoms and cortisol levels; however, these pathways require further empirical validation using more robust analytical models [165].

Furthermore, the evidence is predominantly derived from high-income countries, which limits the generalizability of findings to lower-income contexts, where age-based discrimination may be more prevalent. Overall, there is limited understanding of how ageism operates within dynamic everyday interactions, as well as a scarcity of studies addressing its influence at the institutional level. Consequently, further research is needed to integrate these multiple levels of analysis to more comprehensively understand the impact of ageism on cognition and aging [165].

#### 6.3.2. Public Policies

Public policies can foster ageism as an unintended consequence. Singapore implemented the Generation Pioneer Policy (GPP) in 2013, allocating financial resources for outpatient healthcare for people aged 65 and over. It was found that positive stereotypes related to old age decreased and negative stereotypes increased, possibly due to the increased prevalence of the medicalization of aging [166].

Public policies that highlight the gerontological dividend, that is, that also promote social participation, in addition to improving the representation of old age, can contribute to older adults participating in more activities, some of which can maintain cognitive performance [167].

There are several strategies to promote the social participation of older adults. Laws amended to recognize older adults as subjects of rights, not just recipients of welfare programs. Infrastructure is also being developed to promote social participation. This can include opening centers where volunteering is possible, as well as infrastructure modifications to facilitate transportation and interaction among people. Furthermore, social participation is encouraged through awareness campaigns, support for the creation or strengthening of centers for older adults that promote activities related to learning new skills or implementing mentorship programs, the development of flexible employment policies for older adults, and the creation of forums where people can identify their barriers to participation and design solutions, among other initiatives [155,168,169,170,171].

An umbrella review of fifty-seven systematic reviews analyzed 53 public policies aimed at promoting physical activity. The review identified school policies and infrastructure elements as having the greatest impact. The latter involved interventions that improve aspects of the built environment, such as residential density, mixed land use, sidewalk quality and connectivity, and street redesign to facilitate transportation and cycling. Although most studies have focused on children, their findings may also be transferable to older adults; nevertheless, further research is required to establish the effect on this population [172]. Positive effect has also been reported by media campaigns and multi-component approaches in community interventions [173].

Another umbrella review, which included twelve systematic reviews on the effect of public policies on eating behavior, reported that policies involving food prices (increasing taxes on unhealthy products and subsidies on healthy ones), portion sizes, and food availability in retail establishments promote healthy eating [156]. Currently, it would also be relevant to consider the design of public policies regarding food delivery platforms, which could favor the consumption of healthy or unhealthy foods [157]. Despite the growing body of evidence, there remains a need to investigate the impact of these dietary policies on cognition in older adults.

To reduce pollution, various public policies have been developed. Some consist of incentives, such as subsidies for public transport to make it cheaper and reduce the use of private transport, or for switching from domestic fuels to less harmful ones; closures or restrictions on coal-fired industries or power plants; promotion of the use of nuclear, solar, or wind energy; and stricter vehicle regulations. Several of these strategies may contribute to reducing air pollutants and improving health [174,175,176], with positive effects on cognition [177]. However, evidence on the impact of public policies aimed at reducing air pollution on cognitive functioning in older adults remains limited.

It is important to note that the analysis in this review regarding the macro-level related to public policies represents the evidence that has been most published; however, it should be considered that there are more factors and that they will be different in each country and region.

## 7. Conclusions

This narrative review synthesizes evidence to elucidate the environmental determinants of cognitive aging. We examine neural plasticity and scaffolding as biological mechanisms underlying cognitive maintenance, alongside key modifiable pathways including intellectually stimulating activities, physical exercise, dietary, air pollution, depression, and psychological stress. By integrating environmental data across micro-, meso-, and macro-levels, this review maps these external factors onto established cognitive health pathways. Such a multi-level framework enables a comprehensive conceptualization of cognitive aging as a dynamic emergence resulting from the interaction between biological, behavioral, and environmental processes.

The studies analyzed presented substantial heterogeneity, methodological limitations, and inconsistencies across findings. There was considerable variability in the indicators used to operationalize environmental exposures, including the use of multiple metrics for the same attribute, which limited comparability across studies. Most studies relied on global measures of cognition, restricting the assessment of specific cognitive domains. In addition, some constructs across the micro- and meso-environmental levels were not clearly differentiated, such as loneliness and social networks. The majority of the available evidence originated from high-income countries, limiting the external validity of existing findings. Few studies explicitly examined mediators through which environmental factors may influence cognition, as well as potential moderators, including age, sex, and educational level. Furthermore, most of the evidence was derived from observational designs. Considering these limitations, the following associations between environmental factors and cognition in older adults were identified.

At the micro-environmental level, we find social and physical factors. The social factor can be a motivator for a healthy lifestyle, including intellectually stimulating activities, exercise, and a healthy diet, as well as contribute to the reduction or protection against depressive symptoms. In this sense, the family environment, such as partners, adult children, and grandchildren, is essential for maintaining cognitive function [75,178]. In terms of physical factors, a design that facilitates a healthy lifestyle and avoids air pollution, mainly from fuels such as coal or firewood, can affect cognition [77].

At the meso-environmental level, cognitive health is modulated by community-scale factors, including social interactions, land-use patterns, transportation system, urban design, and the availability of open spaces. Heterogeneous environments that integrate green infrastructure and accessible public realms may facilitate intellectual engagement by fostering diverse social interactions and civic participation, such as volunteering. To maximize cognitive benefits, environmental interventions may need to be culturally and geographically tailored to ensure community uptake and sustained impact. Conversely, proximity to high-traffic corridors or industrial zones may increase exposure to neurotoxic air pollutants, compromising cognitive integrity. Evidence suggests that strategic urban planning focused on nature-based solutions could help alleviate these environmental stressors, offering a plausible pathway for promoting cognitive maintenance [19,108].

In relation to the macro-level environment, public policies and ageism can affect cognition, which are also interconnected. Self-inflicted ageism can discourage older adults from adopting a healthy lifestyle as well as cause depression or stress that also affects cognition. Interpersonal and institutional ageism can limit development opportunities and generate a negative view of old age. Public policies focused solely on the medicalization of aging can lead to undesirable consequences such as increased ageism. Therefore, it is also important to highlight policies that consider older adults as a gerontological asset and the contributions they can make to society [158,160,165].

Several public policies have been developed to improve the lifestyles of the general population and older adults, with potential positive health outcomes. Among the strategies that have been linked to satisfactory results are facilitating access to places or improving infrastructure for people to engage in physical activity; facilitating access to healthy foods and hindering access to unhealthy foods; and promoting various strategies to improve air quality [157,172,176]. However, little is known about how these public policies affect the cognition of older adults.

Available evidence suggests associations between micro-, meso-, and macro-level environmental factors and cognition in ageing. However, this body of literature is predominantly based on observational designs, exhibits substantial methodological and conceptual heterogeneity, and shows inconsistencies in findings, alongside gaps in knowledge. In this context, critical challenges remain for advancing the field.

Underexplored areas: (i) Research on micro- and macro-environmental levels remains limited compared with the meso-level. (ii) The influence of architectural design, whether within individual homes or across the broader built environment, on cognitive functions remains inadequately described. (iii) Additional research is needed in populations from low- and middle-income countries. (iv) The interaction between objective and subjective dimensions of the environment remains poorly understood, encompassing not only structural attributes but also patterns of use and individual preferences.

Methodological and conceptual considerations: (i) To reduce heterogeneity, the adoption of standardized taxonomic frameworks may improve consistency and facilitate the accumulation of evidence. (ii) Assessing specific cognitive functions, particularly those sensitive to aging—such as working memory and episodic memory—would enhance interpretability. (iii) Studies incorporating moderators, such as the key pathways proposed in this work, as well as mediators, using appropriate statistical techniques, should be encouraged. (iv) Experimental and quasi-experimental studies are needed to strengthen causal inference and directly evaluate the impact of environmental modifications on cognitive function.

This review delineates critical environmental factors and their corresponding key pathways, providing a conceptual framework to guide future research across multiple environmental scales. By identifying these determinants, this work seeks to inform research agendas aimed at evaluating how environmental modifications may support cognitive health, autonomy, and overall well-being within the paradigm of healthy aging. Ultimately, the evidence synthesized herein suggests plausible factors for exploring targeted interventions that account for the diverse and evolving contexts of the aging population.

## Figures and Tables

**Table 1:** Environmental characteristics across micro-, meso- and macro-levels associated with protective and adverse cognitive outcomes in older adults.

Environment	Protective Factors for Cognition	Adverse Factors Affecting Cognition
Micro-level (family)	Marital quality Mutual support with adult children Spaces that foster social interaction	Widowhood Loneliness Pollution Extreme temperatures
Meso-level (community)	Social interactions in community	
	Larger network size Higher frequency of contact Participation in social activities	Social exclusion
	Land use patterns	
	Proximity to a community center Access to active leisure activities Access to healthy food	Remoteness from everyday services Access to unhealthy food
	Transport system	
	Improved connectivity and street integration Walkability	Areas with few intersections Social isolation Living near traffic
	Environmental design	
	Perception of safe and clean environments	Perception of danger and physical disorder
	Open spaces	
	Green or blue areas Trees with wide canopies	
Macro-level (society)	Considering the older adults as a “gerontological dividend” Public policies to increase Intellectually stimulating activities, physical activity, healthy food consumption	Viewing the older adults as an “ageing tsunami” Absence or inefficiency of public policies that promote healthy aging

## Data Availability

No new data were created or analyzed in this study.

## Abbreviations

The following abbreviations are used in this manuscript:

MCI	Mild Cognitive Impairment
PM	Particulate Matter
NO2	Nitrogen Dioxide
ROS	Reactive Oxygen Species
